# Supplementary material for: kinGEMs: A robust and scalable framework for resource-constraint models through stochastic tuning of deep learning-predicted kinetic parameters
Source: PLoS Comput Biol. 2026 Nov 6;22(11):e1014881. doi: 10.1371/journal.pcbi.1014881 (PMC13759144; doi:10.1371/journal.pcbi.1014881)
Supplement: Supplementary file 1 [file pcbi.1014881.s001.pdf]

Appendix A kinGEMs Pipeline

The kinGEMs pipeline is constructed to take in a pre-existing GEM as input along with a config file (in JSON format) with specifications for modeling parameters like total enzyme concentration as well as simulated annealing hyperparameters. The pipeline starts by reading the SBML file of the input GEM and extracting the SMILES representations of all the metabolites in the model through multiple SMILES-retrieval databases (Steps 1 and 2 in Fig A1). The pipeline also retrieves all the enzyme sequences in the GEM through the available gene IDs for all the reactions.

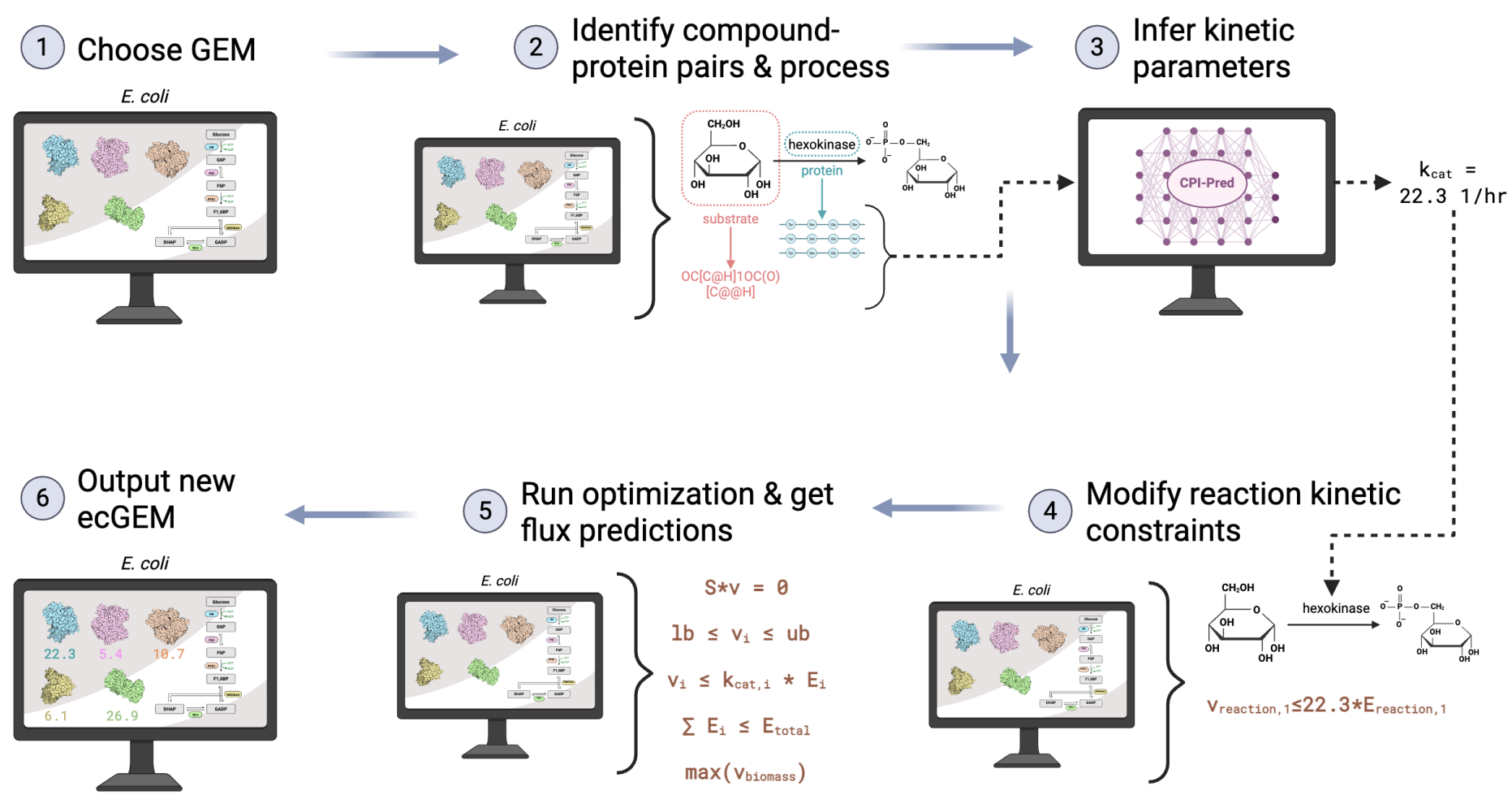

Fig A1: **kinGEMs Process Overview. 1** The kinGEMs pipeline starts with a pre-existing GEM as an input. **2** The compound-protein pairs in the entire GEM are then identified and processed. The SMILES representation of all the metabolites in the cell are obtained while the genes are used to identify and retrieve the corresponding protein sequence. **3** These compound-protein pair SMILES and sequences are sent to CPI-Pred where model inference is run to predict k_{cat} values for all the pairs. **4** The k_{cat} values are processed and mapped to specific reaction constraints through the GPR rules. **5** The optimization problem is run with all corresponding constraints and flux predictions are obtained. Simulated annealing is also run in this step. **6** The final annotated ecGEM is outputted and can be used for further downstream analyses.

The SMILES and protein sequence of every metabolite-enzyme pair is sent to the CPI-Pred model for inference. The model predicts k_{cat} values for each pair with an associated standard deviation value (Step 3 in Fig A1). The standard deviation value is

a product of obtaining five different predictions from five separate cross-validated CPI-Pred models. The pipeline then modifies the reaction constraints with the obtained k_{cat} values through extracting and parsing the gene-protein-reaction rules in the GEM. This process is discussed in more detail in Algorithm 1.

Following the set up of the optimization problem with all the constraint conditions, the pipeline utilizes the Pyomo optimization framework [1] to run a linear programming problem with a maximization objective for the biomass (Step 5 in Fig A1). There are also intermediate optional steps pertaining to the simulated annealing process discussed in the Methods section. The pipeline then saves these newly built ecGEMs, which can be used in other types of analyses.

Algorithm 1. Deriving enzyme-capacity reaction constraints from GPR rules (DNF-based).

Require: Genome-scale model with reactions $\mathcal{R}$, genes $\mathcal{G}$, and GPR strings $\{\text{GPR}_j\}_{j\in\mathcal{R}}$; kinetic database $k_{\text{cat}}(j,g)$ (possibly multiple values per pair); decision variables fluxes v_j and enzyme allocations E_g.
Ensure: A set of linear enzyme-capacity constraints linking v_j to E_g according to each reaction's GPR logic.

Preprocessing: tokenize and convert each GPR to disjunctive normal form (DNF).
for all $j \in \mathcal{R}$ **do**
    if GPR_j is empty **then**
        continue
    end if
    tokens $\leftarrow$ TOKENIZE(GPR_j) ▷ parentheses, AND/OR, gene IDs
    $\mathcal{C}_j \leftarrow$ PARSETODNF(tokens)
    DEDUPLICATE and SORT genes within each clause in $\mathcal{C}_j$
end for

Constraint generation: map DNF structure to enzyme-capacity inequalities.
for all $j \in \mathcal{R}$ such that $\mathcal{C}_j \neq \emptyset$ **do**
    if $|\mathcal{C}_j| = 1$ **and** $|\mathcal{C}_j[1]| = 1$ **then**
        let g be the only gene in the sole clause
        if $k_{\text{cat}}(j,g)$ exists **then**
            $\kappa_{j,g} \leftarrow \max k_{\text{cat}}(j,g)$
            add constraint: $v_j \leq \kappa_{j,g} E_g$
        end if
    else if $|\mathcal{C}_j| = 1$ **and** $|\mathcal{C}_j[1]| > 1$ **then**
        let c be the sole clause and $\mathcal{K} \leftarrow \bigcup_{g\in c} k_{\text{cat}}(j,g)$
        if $\mathcal{K} \neq \emptyset$ **then**
            $\kappa_{j,c} \leftarrow \max(\mathcal{K})$
            for all $g \in c$ **do**
                add constraint: $v_j \leq \kappa_{j,c} E_g$
            end for
        end if
    else if $|\mathcal{C}_j| > 1$ **and** $\forall c \in \mathcal{C}_j : |c| = 1$ **then**
        $\mathcal{T} \leftarrow \emptyset$
        for all $c \in \mathcal{C}_j$ **do**
            let g be the only gene in c
            if $k_{\text{cat}}(j,g)$ exists **then**
                $\kappa_{j,g} \leftarrow \max k_{\text{cat}}(j,g)$
                $\mathcal{T} \leftarrow \mathcal{T} \cup \{\kappa_{j,g} E_g\}$
            end if

        end for
        if $\mathcal{T} \neq \emptyset$ **then**
            add constraint: $v_j \leq \sum_{t \in \mathcal{T}} t$
        end if
    else
        $\mathcal{T} \leftarrow \emptyset$
        for all $c \in \mathcal{C}_j$ **do**
            $\mathcal{K}_c \leftarrow \bigcup_{g \in c} k_{\text{cat}}(j, g)$
            if $\mathcal{K}_c = \emptyset$ **then**
                continue
            end if
            if $|c| > 1$ **then**
                $\bar{\kappa}_{j,c} \leftarrow \frac{1}{|\mathcal{K}_c|} \sum_{k \in \mathcal{K}_c} k$
                $t_c \leftarrow \bar{\kappa}_{j,c} \frac{1}{|c|} \sum_{g \in c} E_g$
            else
                let g be the only gene in c
                $\kappa_{j,g} \leftarrow \max \mathcal{K}_c$
                $t_c \leftarrow \kappa_{j,g} E_g$
            end if
            $\mathcal{T} \leftarrow \mathcal{T} \cup \{t_c\}$
        end for
        if $\mathcal{T} \neq \emptyset$ **then**
            add constraint: $v_j \leq \sum_c t_c$
        end if
    end if
end for

Optional: promiscuous-enzyme coupling across reactions.
for all $g \in \mathcal{G}$ **do**
    $\mathcal{U} \leftarrow \emptyset$
    for all $j \in \mathcal{R}$ such that g appears in some $c \in \mathcal{C}_j$ **do**
        for all $k \in k_{\text{cat}}(j, g)$ **do**
            $\mathcal{U} \leftarrow \mathcal{U} \cup \left\{ \frac{v_j}{k} \right\}$
        end for
    end for
    if $\mathcal{U} \neq \emptyset$ **then**
        add constraint: $\sum_{u \in \mathcal{U}} u \leq E_g$
    end if
end for

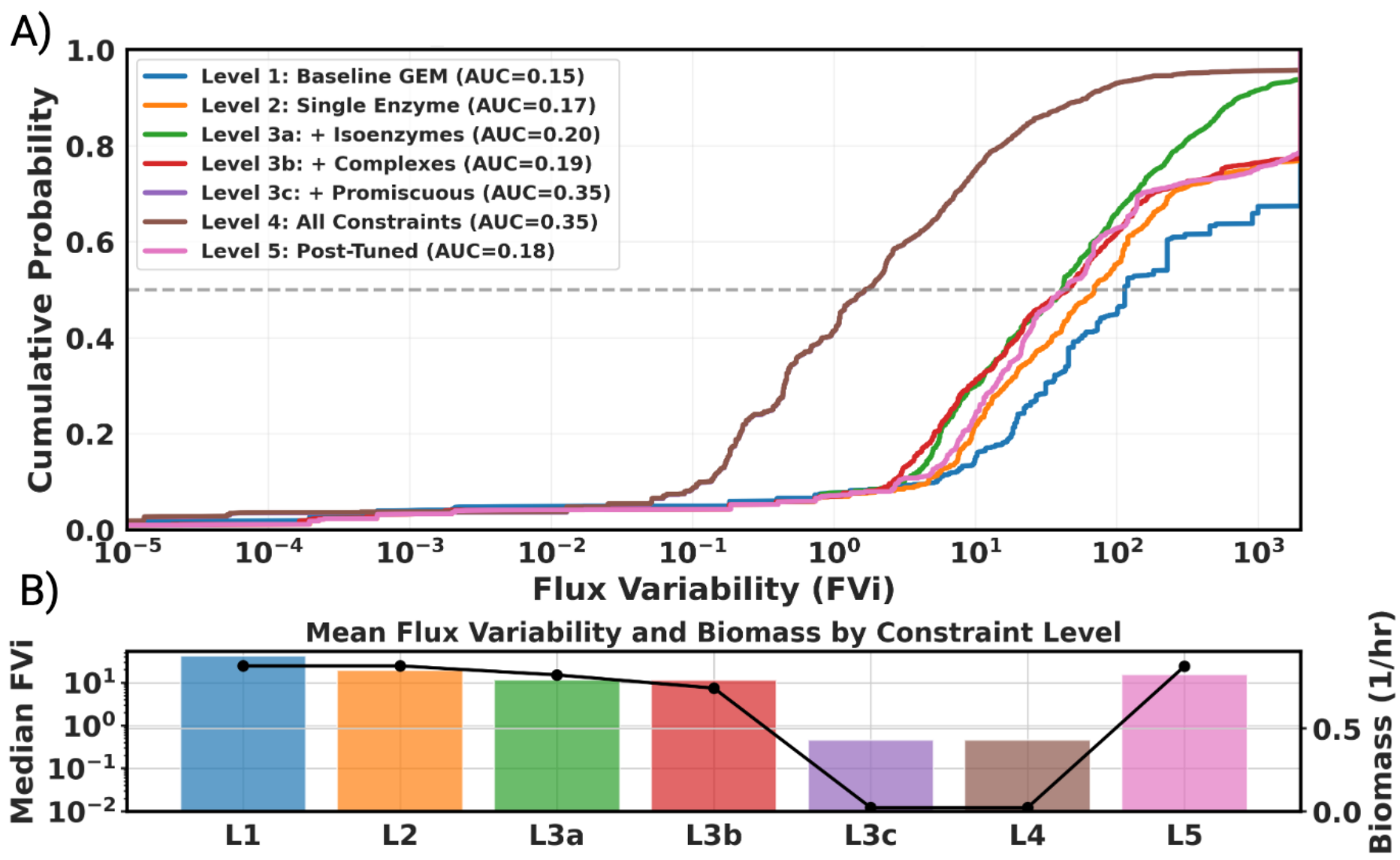

Fig A2: Flux variability ablation study. Progressive integration of enzyme constraints systematically reduces the feasible **non-optimal** flux solution space. **A)** Cumulative distribution probability of flux variability (FVi) across all reactions for each constraint level. Leftward shifts of the curves indicate tighter feasible flux ranges for an increasing fraction of reactions as enzymatic constraints are added. The dashed line denotes the median FVi. Here, $\text{FVi} = v_i^{\max} - v_i^{\min}$ denotes the flux variability of reaction i. AUC here denotes area under the curve, where a higher value indicates greater proportion of reactions with low flux variability. *The level 3c line in the plot overlaps with Level 4, and is therefore not clearly visible in the plot. **B)** Median flux variability (bars, left axis) and corresponding optimal biomass flux (points, right axis) for each constraint level. While stronger constraints substantially reduce flux variability, they introduce trade-offs with achievable growth. Post hoc tuning of kinetic parameters (L5) restores biomass production while maintaining lower variability than L1.

Appendix B Tuning ecGEMs Towards Experimental Alignment

As part of the kinGEMs pipeline, we present a simulated annealing algorithm that tunes the ML-predicted k_{cat} values to better align them with experimental observations. In all the results presented in this paper, maximization of biomass is used as the objective function value. However, the simulated annealing framework allows for the incorporation any experimental data (fluxomics, metabolomics, proteomics, etc.) and the alignment of these ecGEMs with biological reality.

Algorithm 2 Simulated Annealing for Enzyme k_{cat} Optimization

Require: Metabolic model $\mathcal{M}$, processed kinetic data $\mathcal{D}$, biomass reaction r_b, target biomass B^*, gene sequences $\mathcal{G}$, initial temperature T_0, cooling rate α, minimum temperature $T_{\min}$, maximum iterations $I_{\max}$
Ensure: Optimized k_{cat} dictionary, best biomass solution

```
Compute molecular weights for all genes in 𝒢
Run initial enzyme-constrained FBA to obtain biomass B_0
if B_0 ≤ 0 then
    return empty result (initial optimization failed)
end if
Select top N enzymes by mass contribution
Initialize current solution k^(0) using original k_cat
Set best solution k* ← k^(0), B* ← B_0
T ← T_0, iteration i ← 1
while T > T_min and i < I_max and B < B* do
    Select random subset of enzymes to perturb
    for each selected enzyme (r, g) do
        Generate neighbor k'_{r,g} using biased Gaussian perturbation
        Clamp k'_{r,g} within biologically valid bounds
        Update 𝒟 with k'_{r,g}
    end for
    Run enzyme-constrained FBA with updated 𝒟
    if optimization fails then
        Reject proposal and continue
    end if
    Compute acceptance probability
```

$$p = \begin{cases} 1, & \text{if } B_{\text{new}} > B \\ \exp\left(\dfrac{B_{\text{new}} - B}{T}\right), & \text{otherwise} \end{cases}$$

```
    if p > Uniform(0, 1) then
        Accept new solution k ← k'
        B ← B_new
        if B > B* then
            k* ← k
            B* ← B
        end if
    end if
    T ← αT
    i ← i + 1
end while
return optimized k_cat values k* and best biomass B*
```

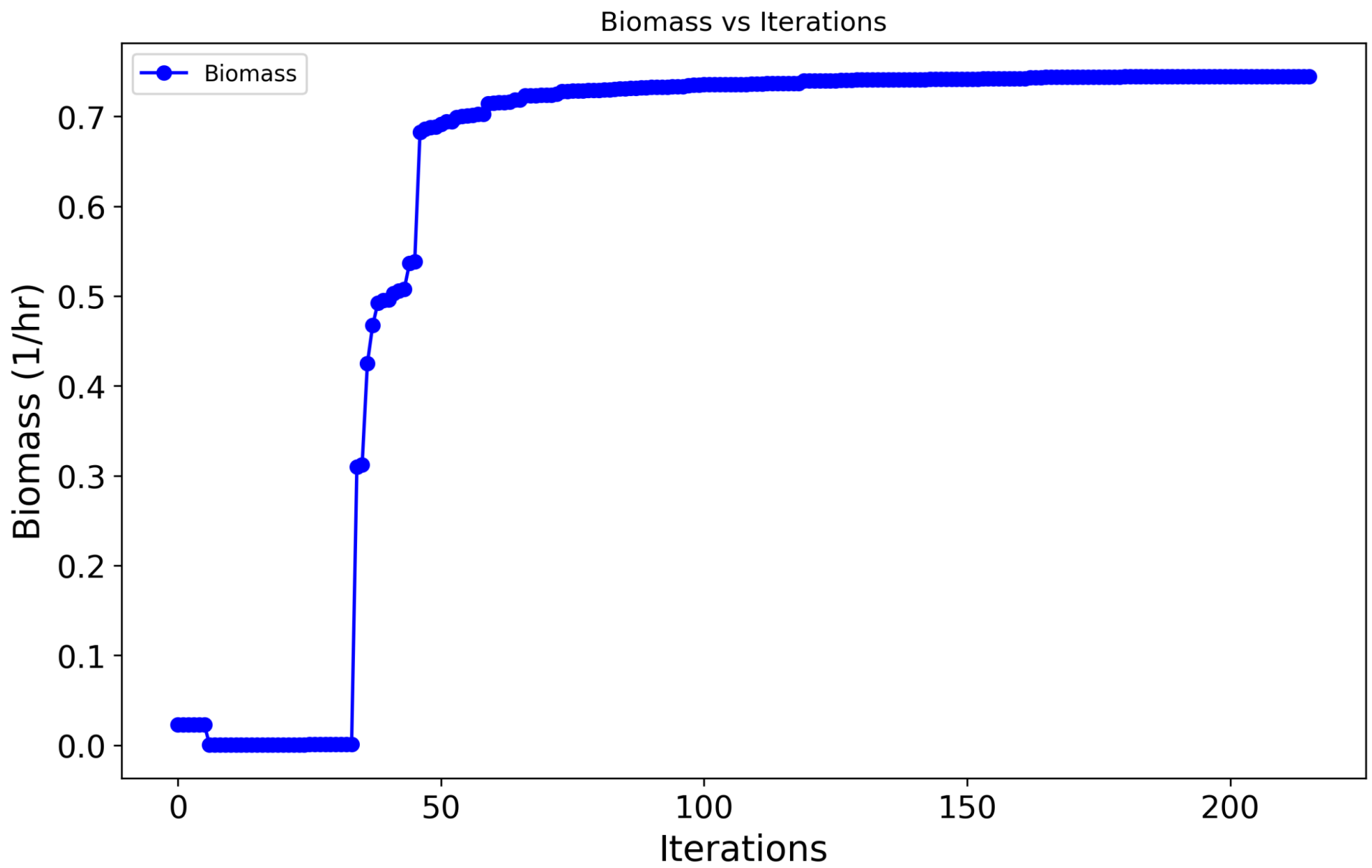

Fig B3: Optimization of biomass production during simulated annealing of kinetic parameters. Biomass objective value as a function of simulated annealing iterations. Early iterations exhibit low or near-zero growth, followed by a rapid transition to feasible growth states as constraints are relaxed. Subsequent iterations show gradual improvement and stabilization of biomass, indicating convergence of the optimization process toward a high-fitness kinetic parameter configuration.

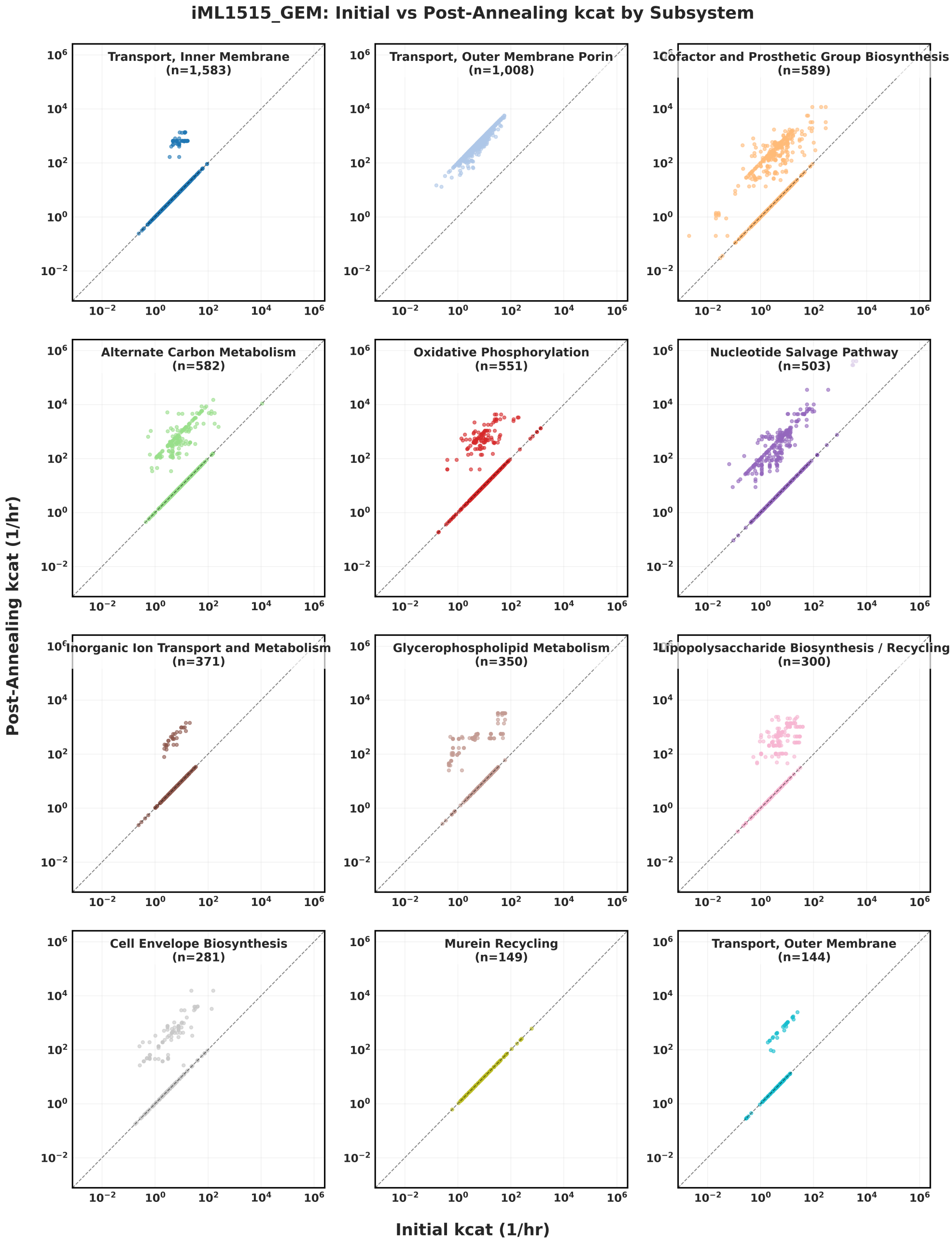

Fig B4: Subsystem-specific shifts in catalytic rate constants (k_{cat}) following simulated annealing optimization. Scatter plots highlighting the shifts of k_{cat} values per subsystem in *E. coli*'s (iML1515) metabolism. Outer membrane porin transport reactions reveal consistent upward bias in the simulated annealing process. Note that there about 5,000 data points with unknown or other smaller subsystems that are excluded from this figure.

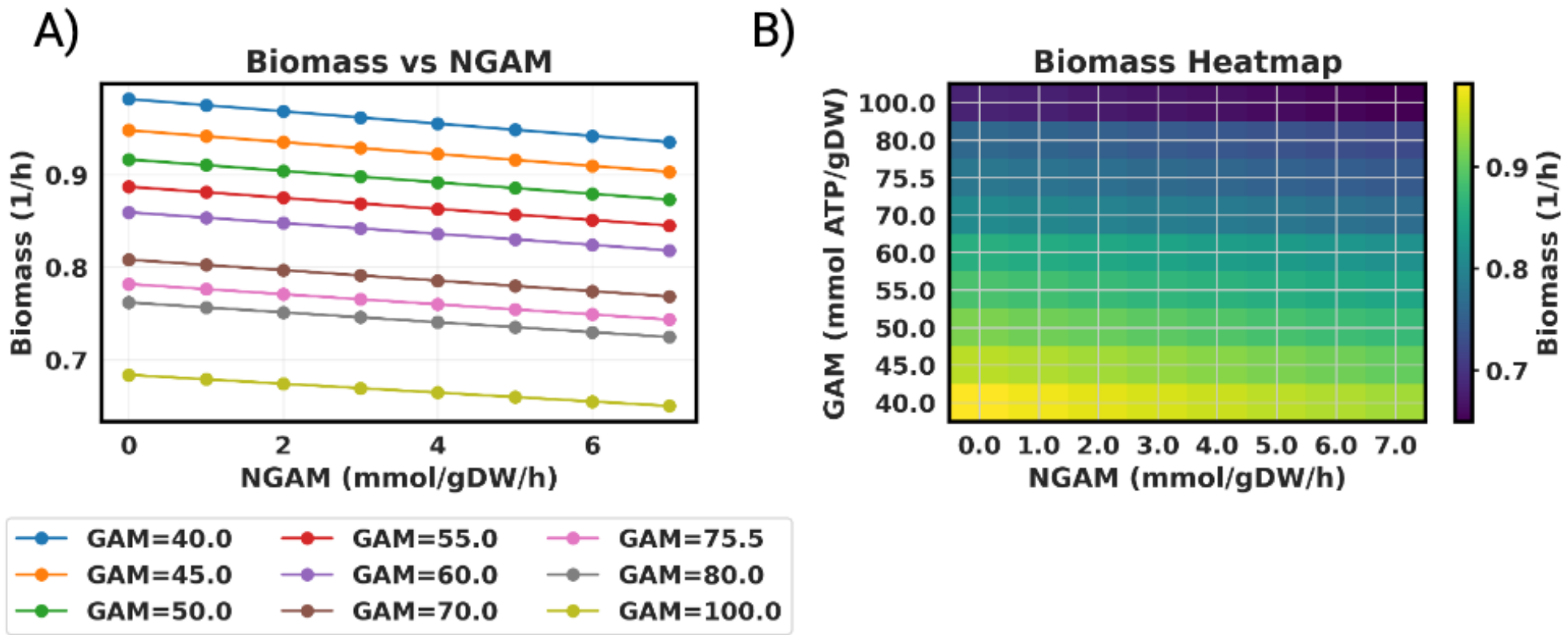

Fig B5: Sensitivity of predicted growth rate to growth-associated (GAM) and non-growth-associated (NGAM) maintenance parameters in *E. coli* iML1515. **(A)** Biomass production rate as a function of NGAM across different fixed GAM values. Increasing NGAM leads to a monotonic decrease in biomass for all GAM settings, while higher GAM values uniformly shift biomass predictions downward, indicating increased energetic cost of growth. **(B)** Heatmap summarizing biomass production across the GAM–NGAM parameter space. Biomass is highest in regions with low GAM and NGAM and decreases smoothly as maintenance energy requirements increase, illustrating the coupled impact of growth and maintenance energy demands on predicted fitness.

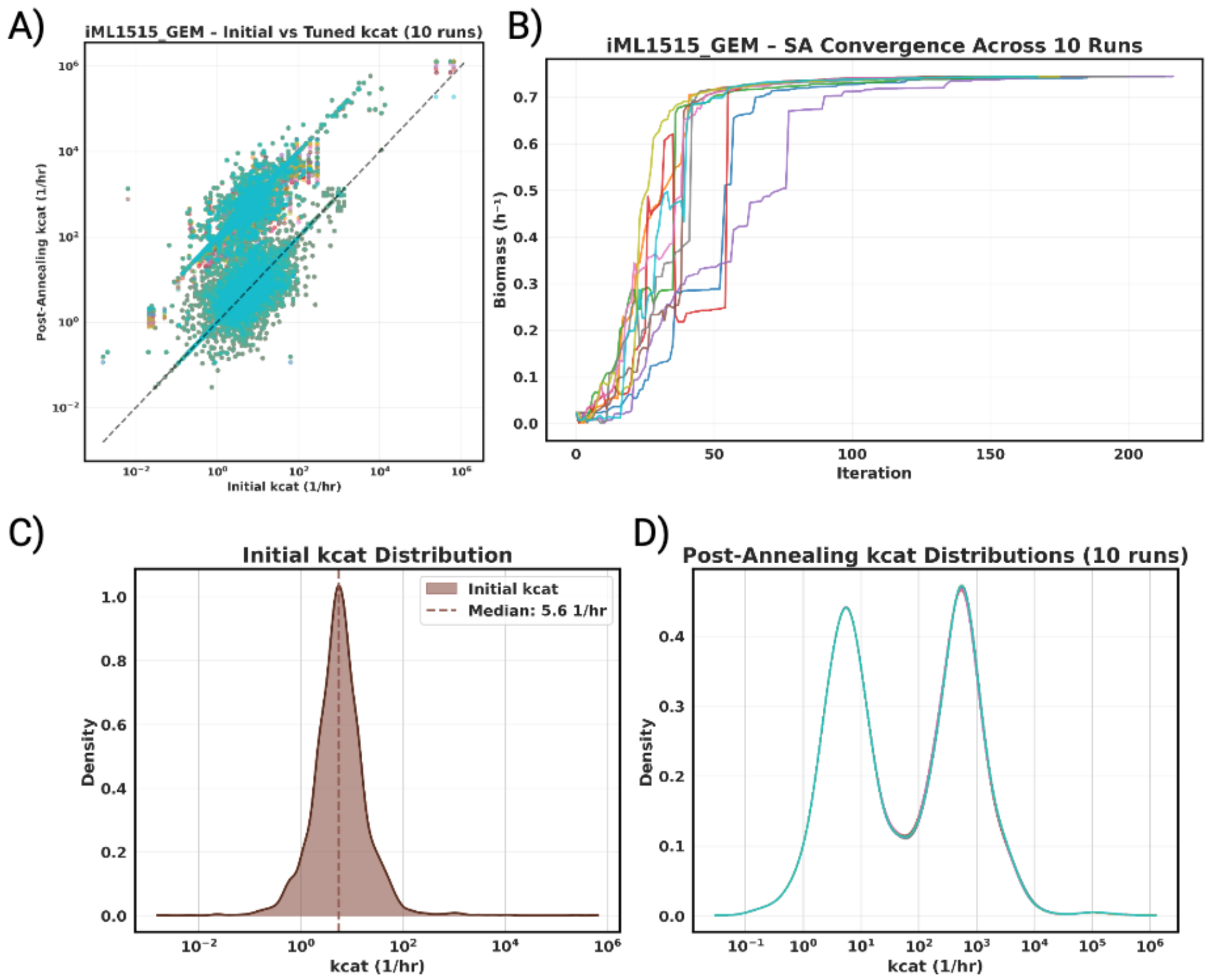

Fig B6: Reproducibility and convergence of simulated annealing optimization across 10 independent runs in *E. coli* iML1515. **(A)** Scatter plot comparing initial and post-annealing k_{cat} values across all 10 runs. The dashed line indicates no change ($y = x$). The majority of points lie above the diagonal, indicating a consistent upward shift in catalytic rates across seeds, with high reproducibility in the direction and magnitude of perturbations. **(B)** Biomass production as a function of simulated annealing iteration across all 10 runs. Despite different random seeds, all runs converge to similar biomass values (0.75 h^{-1}) within approximately 50-100 iterations, demonstrating robust convergence behavior independent of initialization. **(C)** Initial k_{cat} distribution prior to simulated annealing, showing a unimodal distribution with a median of 5.6 h^{-1} concentrated at low turnover rates. **(D)** Post-annealing k_{cat} distributions across all 10 runs (overlaid). All runs produce nearly identical bimodal distributions with a rightward shift relative to the initial distribution, indicating that the optimization consistently identifies similar regions of the kinetic parameter space regardless of random seed.

B.1 Sparsity Sweep

As part of the kinGEMs pipeline design, we additionally evaluated how the size of the enzyme perturbation pool (the number of top-by-mass genes simulated annealing is eligible to perturb per iteration ($n\_top\_enzymes$)) affects feasibility, proteomics agreement, and interpretability. We compared $n\_top\_enzymes \in \{5, 10, 20, 50, 500\}$ (CPI-Pred initialization, 8 growth conditions × 4 replicates; Fig B7), spanning a sparse (GECKO/ECMpy-style) regime to the broad (DLKcat-style) regime used throughout the primary results in this study.

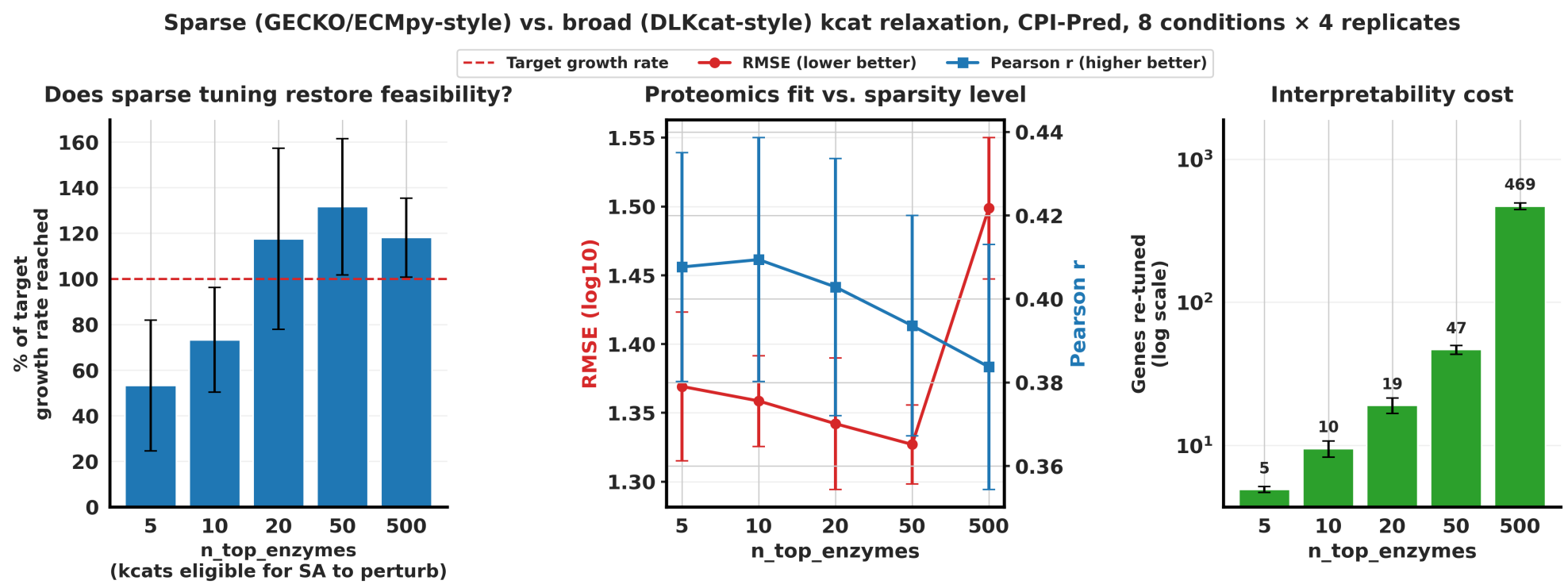

Fig B7: Sparse vs. broad k_{cat} relaxation: effect of perturbation pool size on feasibility, proteomics fit, and interpretability. Comparison of restricting simulated annealing to perturb only the top-n enzymes by mass contribution ($n \in \{5, 10, 20, 50, 500\}$), using CPI-Pred-initialized $k_{cat}s$ across 8 growth conditions with 4 replicates each. **Left:** Percentage of the target growth rate reached after tuning (dashed line = 100%); very small pools ($n = 5$, $n = 10$) fail to reliably restore feasible growth, while $n \geq 20$ consistently meets or exceeds the target. **Center:** Proteomics fit (RMSE, red; Pearson r, blue) against Schmidt et al. proteomics, as a function of pool size. RMSE improves monotonically from $n = 5$ to $n = 50$, then degrades sharply at $n = 500$ (the broad pool used elsewhere in this study); Pearson r shows a milder, comparable trend. **Right:** Number of genes actually re-tuned (log scale) at each pool size, illustrating the interpretability cost of broader relaxation. Together, these results indicate that a moderate perturbation budget ($n \approx$ 20-50) achieves comparable or better feasibility and proteomics accuracy than the broad ($n = 500$) pool used for the primary results in this study, at substantially lower interpretability cost.

Appendix C MFA Experimental Comparison

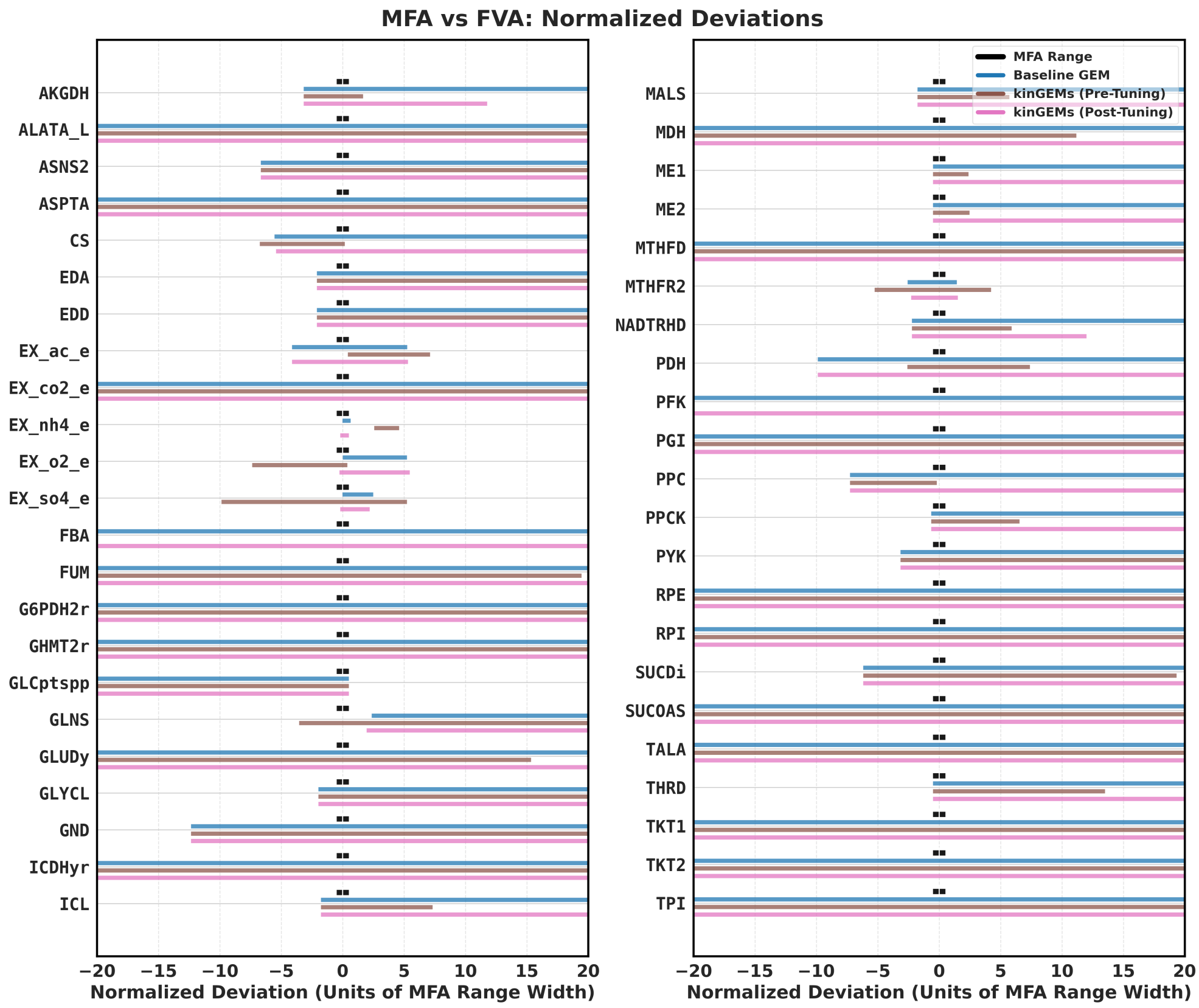

Fig C8: FVA Results Normalized Against Experimental MFA Values. Comparison of experimentally measured ^{13}C MFA flux ranges (black) with model-predicted feasible flux ranges obtained via FVA for all available reactions. Colored horizontal bars indicate FVA ranges for the baseline GEM (blue), kinGEMs before kinetic tuning (brown), and kinGEMs after kinetic tuning (pink). Enzyme constraints substantially narrow feasible flux ranges relative to the baseline model, bringing predictions closer to experimentally observed values. Some reactions have missing kinGEMs (Pre-Tuning) FVA ranges because the optimization failed to find solutions for the reactions at that formulation.

Appendix D Supplementary Proteomics and Phenotype Validation

This appendix provides supplementary detail supporting the proteomics and phenotypic accuracy analyses presented in Section 3.2, using the absolute quantitative

Table C1: Mapping between ^{13}C MFA for intracellular and exchange reactions [2] and iML1515 model reactions.

^{13}C MFA ID	^{13}C MFA Reaction	iML1515 ID	iML1515 Reaction
1	Gluc.Ext + PEP → G6P + Pyr	EX_glc__D_e	glc__D_e ↔
2	G6P ↔ F6P (net)	PGI	g6p_c ↔ f6p_c
3	F6P + ATP → FBP + ADP	PFK	atp_c + f6p_c → adp_c + fdp_c + h_c
4	FBP ↔ DHAP + GAP (net)	FBA	fdp_c ↔ dhap_c + g3p_c
5	DHAP ↔ GAP (net)	TPI	dhap_c ↔ g3p_c
8	PEP + ADP → Pyr + ATP	PYK	adp_c + h_c + pep_c → atp_c + pyr_c
9	G6P + NADP → 6PG + NADPH	G6PDH2r	g6p_c + nadp_c ↔ 6pgl_c + h_c + nadph_c
10	6PG + NADP → Ru5P + CO2 + NADPH	GND	6pgc_c + nadp_c → co2_c + nadph_c + ru5p__D_c
11	Ru5P ↔ X5P (net)	RPE	ru5p__D_c ↔ xu5p__D_c
12	Ru5P ↔ R5P (net)	RPI	r5p_c ↔ ru5p__D_c
14	F6P ↔ E4P + E-C2 (net)	TKT2	e4p_c + xu5p__D_c ↔ f6p_c + g3p_c
15	S7P ↔ R5P + E-C2 (net)	TKT1	r5p_c + xu5p__D_c ↔ g3p_c + s7p_c
16	F6P ↔ GAP + E-C3 (net)	TALA	g3p_c + s7p_c ↔ e4p_c + f6p_c
18	6PG → KDPG	EDD	6pgc_c → 2ddg6p_c + h2o_c
19	KDPG → GAP + Pyr	EDA	2ddg6p_c → g3p_c + pyr_c
20	Pyr + NAD → AcCoA + CO2 + NADH	PDH	coa_c + nad_c + pyr_c → accoa_c + co2_c + nadh_c
21	AcCoA + OAC → Cit	CS	accoa_c + h2o_c + oaa_c → cit_c + coa_c + h_c
23	ICit + NADP ↔ AKG + CO2 + NADPH (net)	ICDHyr	icit_c + nadp_c ↔ akg_c + co2_c + nadph_c
24	AKG + NAD → SucCoA + CO2 + NADH	AKGDH	akg_c + coa_c + nad_c → co2_c + nadh_c + succoa_c
25	SucCoA + ADP + Pi ↔ Suc + ATP (net)	SUCOAS	atp_c + coa_c + succ_c ↔ adp_c + pi_c + succoa_c
26	Suc + FAD ↔ Fum + FADH2 (net)	SUCDi	q8_c + succ_c → fum_c + q8h2_c
27	Fum ↔ Mal (net)	FUM	fum_c + h2o_c ↔ mal__L_c
28	Mal + NAD ↔ OAC + NADH (net)	MDH	mal__L_c + nad_c ↔ h_c + nadh_c + oaa_c
29	ICit ↔ Glyox + Suc (net)	ICL	icit_c → glx_c + succ_c
30	AcCoA + Glyox → Mal	MALS	accoa_c + glx_c + h2o_c → coa_c + h_c + mal__L_c
31	Mal + NADP → Pyr + CO2 + NADPH	ME2	mal__L_c + nadp_c → co2_c + nadph_c + pyr_c
32	Mal + NAD → Pyr + CO2 + NADH	ME1	mal__L_c + nad_c → co2_c + nadh_c + pyr_c
33	PEP + CO2 → OAC + Pi	PPC	co2_c + h2o_c + pep_c → h_c + oaa_c + pi_c
34	OAC + ATP → PEP + CO2 + ADP	PPCK	atp_c + oaa_c → adp_c + co2_c + pep_c
36	AKG + NADPH + NH3 → Glu + NADP	GLUDy	glu__L_c + h2o_c + nadp_c ↔ akg_c + h_c + nadph_c + nh4_c
37	Glu + ATP + NH3 → Gln + ADP + Pi	GLNS	atp_c + glu__L_c + nh4_c → adp_c + gln__L_c + h_c + pi_c
40	OAC + Glu → Asp + AKG	ASPTA	akg_c + asp__L_c ↔ glu__L_c + oaa_c
41	Asp + NH3 + 2 ATP → Asn + 2 ADP + 2 Pi	ASNS2	asp__L_c + atp_c + nh4_c → amp_c + asn__L_c + h_c + ppi_c
42	Pyr + Glu → Ala + AKG	ALATA_L	akg_c + ala__L_c ↔ glu__L_c + pyr_c
44	Ser + THF ↔ Gly + MEETHF (net)	GHMT2r	ser__L_c + thf_c ↔ gly_c + h2o_c + mlthf_c
45	Gly + THF + NAD ↔ CO2 + MEETHF + NH3 + NADH (net)	GLYCL	gly_c + nad_c + thf_c → co2_c + mlthf_c + nadh_c + nh4_c
46	Thr + NAD → Gly + AcCoA + NADH	THRD	nad_c + thr__L_c → 2aobut_c + h_c + nadh_c
59	MEETHF + NADH → METHF + NAD	MTHFR2	2.0 h_c + mlthf_c + nadh_c → 5mthf_c + nad_c
60	MEETHF + NADP → FTHF + NADPH	MTHFD	mlthf_c + nadp_c ↔ methf_c + nadph_c
63	NADH + NADP ↔ NADPH + NAD (net)	NADTRHD	nad_c + nadph_c → nadh_c + nadp_c
65	Ac → Ac.Ext	EX_ac_e	ac_e →
66	CO2 → CO2.Ext	EX_co2_e	co2_e ↔
67	O2.Ext → O2	EX_o2_e	o2_e ↔
68	NH3.Ext → NH3	EX_nh4_e	nh4_e ↔
69	SO4.Ext → SO4	EX_so4_e	so4_e ↔
70	0.488 Ala + 0.281 Arg + 0.229 Asn + 0.229 Asp + 0.087 Cys + 0.25 Glu + 0.25	BIOMASS_Ec_iML1515_core_75p37M	0.000223 10fthf_c + 2.6e-05 2fe2s_c + 0.000223 2ohph_c + 0.00026 4fe4s_c + 0.514 ala__L_c + 0.296 arg__L_c + 0.241 asn__L_c + ... + 0.423 val__L_c + 0.000341 zn2_c → 75.4 adp_c + 75.4 h_c + 75.4 pi_c + 0.774 ppi_c

Table C2: Wilcoxon signed-rank tests comparing Baseline GEM against kinGEMs (post-tuning) and kinGEMs (pre-tuning) for mean-to-mean flux distance and FVA interval width (log-scale). Using glucose-normalized fluxes (rescaled to glucose uptake = 100). Effect size is reported as rank-biserial correlation r; $^{*}p < 0.001$.

**Metric**	**Comparison**	**Median (Baseline)**	**Median (model)**	n	p**-value**	r
Mean-to-mean distance	vs. kinGEMs (post-tuning)	48.54	41.59	46	$2.77 \times 10^{-4*}$	0.929
	vs. kinGEMs (pre-tuning)	48.54	14.19	46	$2.06 \times 10^{-4*}$	0.836
FVA interval width (log-scale)	vs. kinGEMs (post-tuning)	380.23	292.93	45	$4.33 \times 10^{-4*}$	0.813
	vs. kinGEMs (pre-tuning)	380.23	104.97	45	$1.44 \times 10^{-6*}$	0.921

proteomics dataset of Schmidt et al. [3] across 15 growth conditions and 4 k_{cat} initialization sources (CPI-Pred, an independent deep learning predictor CatPred, literature-derived BRENDA values, and randomly sampled log-normal values).

D.1 Growth-rate phenotype agreement

As an additional phenotype-level check, we compared model-predicted growth rate (the FBA objective) against experimentally reported growth rates from Schmidt et al. across all 15 conditions and 4 k_{cat} sources (n=55; Fig D9). Predicted and experimental growth rates showed strong agreement (r = 0.73, MAE = 0.12 h^{-1}). We note this agreement is expected to be strong rather than an independent validation, since simulated annealing actively searches for k_{cat} values that hit the experimental growth rate as its tuning target for each condition; it confirms that the enzyme-mass budget used throughout this study is large enough to reach physiologically realistic growth rates across all 15 conditions tested without hitting an infeasible ceiling, and is presented here for completeness rather than as free out-of-sample evidence of predictive accuracy.

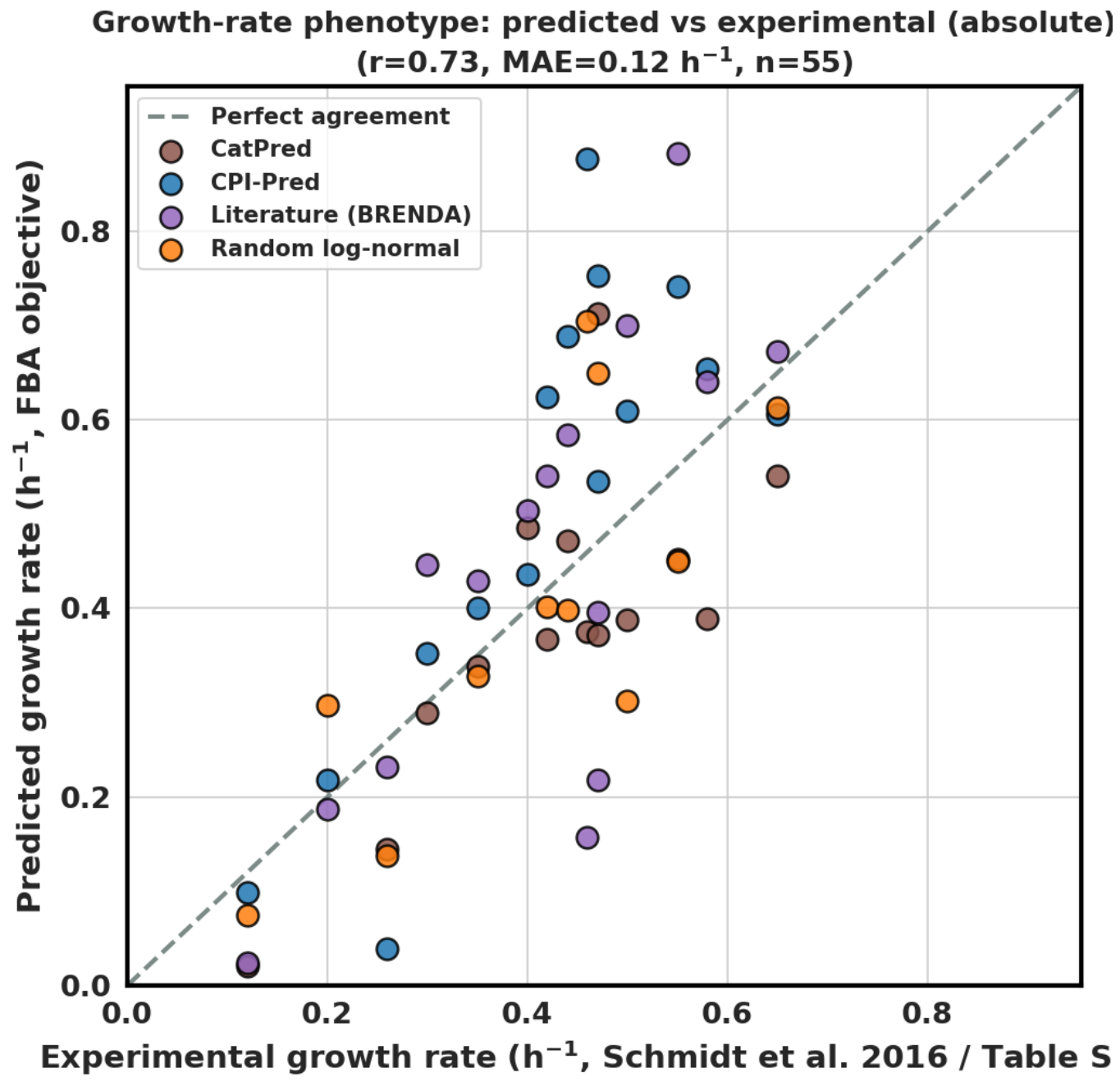

Fig D9: Predicted vs. experimental growth rate across 15 growth conditions and 4 k_{cat} sources. Model-predicted growth rate (FBA objective, y-axis) vs. experimentally reported growth rate (Schmidt et al. 2016, Table S23, x-axis) for all 15 growth conditions and 4 k_{cat} sources ($r = 0.73$, MAE $= 0.12$ h^{-1}, $n = 55$). See Section D.1 for discussion of why this agreement should not be read as independent validation.

D.2 Generalization and Specificity of Simulated-Annealing-Tuned Kinetic Parameters

A central question for any parameter-tuning procedure is whether the tuned parameters capture generalizable, biologically meaningful structure or simply overfit to the specific objective and data used during optimization. We assessed this along two independent axes: whether a k_{cat} vector tuned under one growth condition generalizes to a different condition's medium (Section D.2.2), and whether proteomics-aware tuning targeted at one set of genes generalizes to genes in other, untouched metabolic pathways within the same condition (Section D.2.1). These two checks yield different answers: growth-rate-driven k_{cat} tuning transfers well across growth conditions, while gene-subset-targeted proteomics-aware tuning does not demonstrate reliable transfer to a held-out pathway.

D.2.1 Pathway-level specificity of proteomics-aware tuning

Genes were split by metabolic pathway into a training set (energy metabolism; glycolysis, TCA cycle, pentose phosphate pathway, oxidative phosphorylation, and pyruvate metabolism; the objective genes for the proteomics-aware tuning phase) and a held-out test set (ten amino acid biosynthesis pathways, never touched by proteomics-aware tuning). For each of the 15 growth conditions and 4 k_{cat} sources, we compared RMSE against Schmidt et al. proteomics before and after proteomics-aware tuning, separately for genes in the training pathway and genes in the held-out pathway (Fig D10).

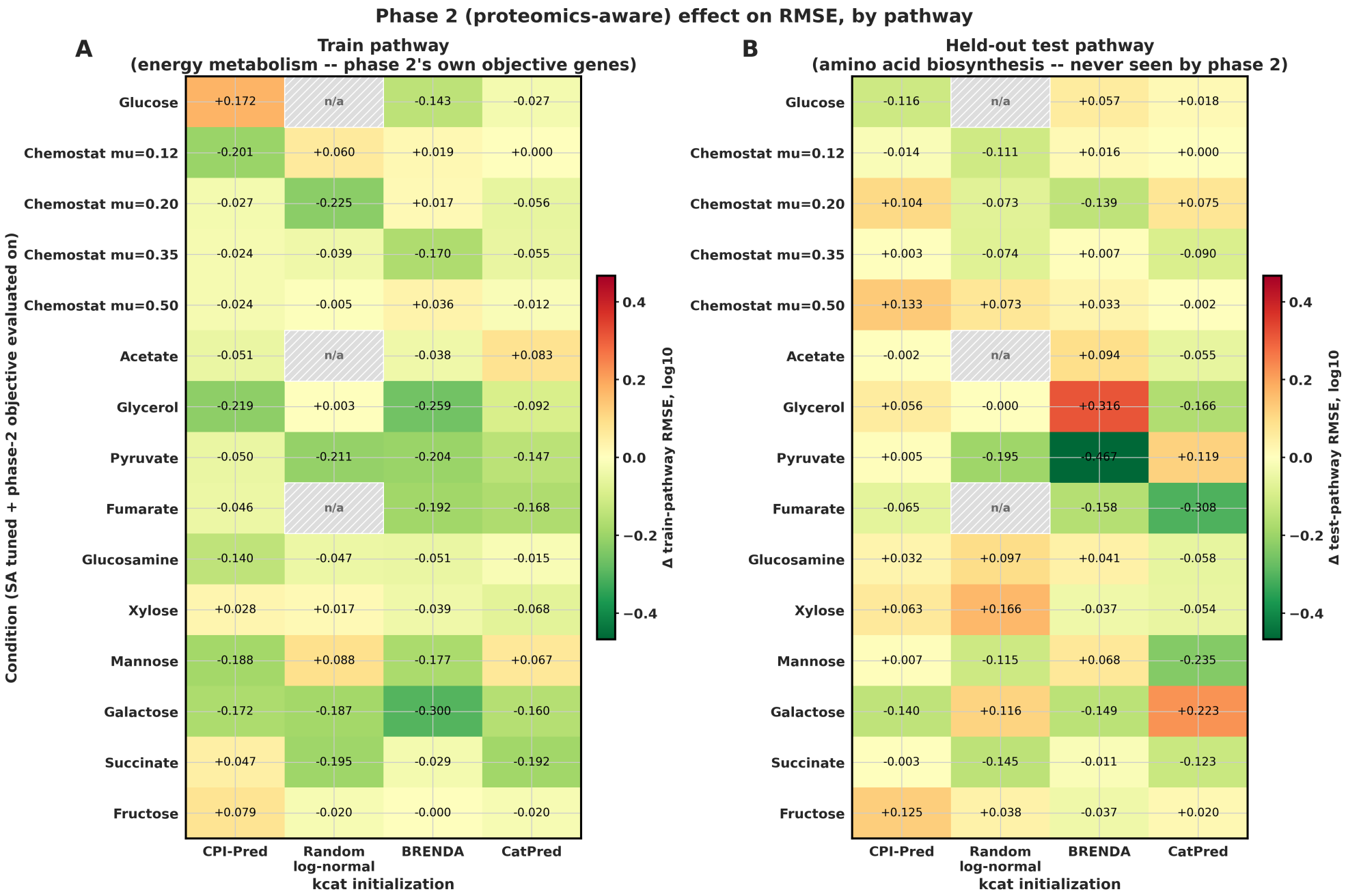

Fig D10: Proteomics-aware tuning generalizes from trained to held-out metabolic pathways. Change in RMSE against Schmidt et al. proteomics following proteomics-aware (Phase 2) tuning, relative to growth-rate-only (Phase 1) tuning, by growth condition (rows) and k_{cat} initialization source (columns). **(A)** Change in RMSE for the training pathway (energy metabolism: glycolysis, TCA cycle, pentose phosphate pathway, oxidative phosphorylation, pyruvate metabolism), the direct objective of Phase 2 tuning. **(B)** Change in RMSE for the held-out test pathway (amino acid biosynthesis, 10 pathways), never included in the Phase 2 objective. Green indicates improved (lower) RMSE after proteomics-aware tuning; orange/red indicates worsened RMSE. Hatched cells (n/a) indicate condition-source combinations for which simulated annealing did not converge to a feasible solution. Panel B's condition-source RMSE changes were not statistically significant in any completed run (95% bootstrap confidence intervals; Methods 5.6.3), and should not be interpreted as evidence that proteomics-aware tuning generalizes to genes outside its direct training objective.

Proteomics-aware tuning reduced RMSE for the training (energy metabolism) pathway in the majority of condition-source combinations (green cells, panel A), as expected given these genes were the direct tuning objective and only proteomics-fit-improving moves are accepted by construction (Methods 5.6.3). The held-out amino acid biosynthesis pathway (panel B) shows a visually mixed pattern of improvement and degradation across condition-source combinations; however, this change was not

statistically significant in any completed run when assessed via 95% bootstrap confidence intervals (percentile method, resampling matched gene pairs; Methods 5.6.3). We therefore do not interpret panel B as evidence that proteomics-aware tuning generalizes to genes outside its direct training objective; the point-estimate RMSE changes shown here should be read alongside this caveat rather than as a standalone indicator of transfer. Cells marked n/a correspond to condition-source combinations for which the underlying simulated annealing run did not converge to a feasible solution.

D.2.2 Condition-level generalization: cross-condition transfer

We additionally tested whether a k_{cat} vector tuned under one growth condition retains its proteomics fit when applied, without further tuning, to a different condition's medium. For each of the 15 conditions and 4 k_{cat} sources, we computed the native (matched-condition) RMSE against Schmidt et al. proteomics, expressed relative to the grand mean RMSE across all condition-source cells (1.541; panel A), and the cross-condition transfer effect, defined as the mean RMSE across all other target conditions minus the native RMSE (panel B; Fig D11).

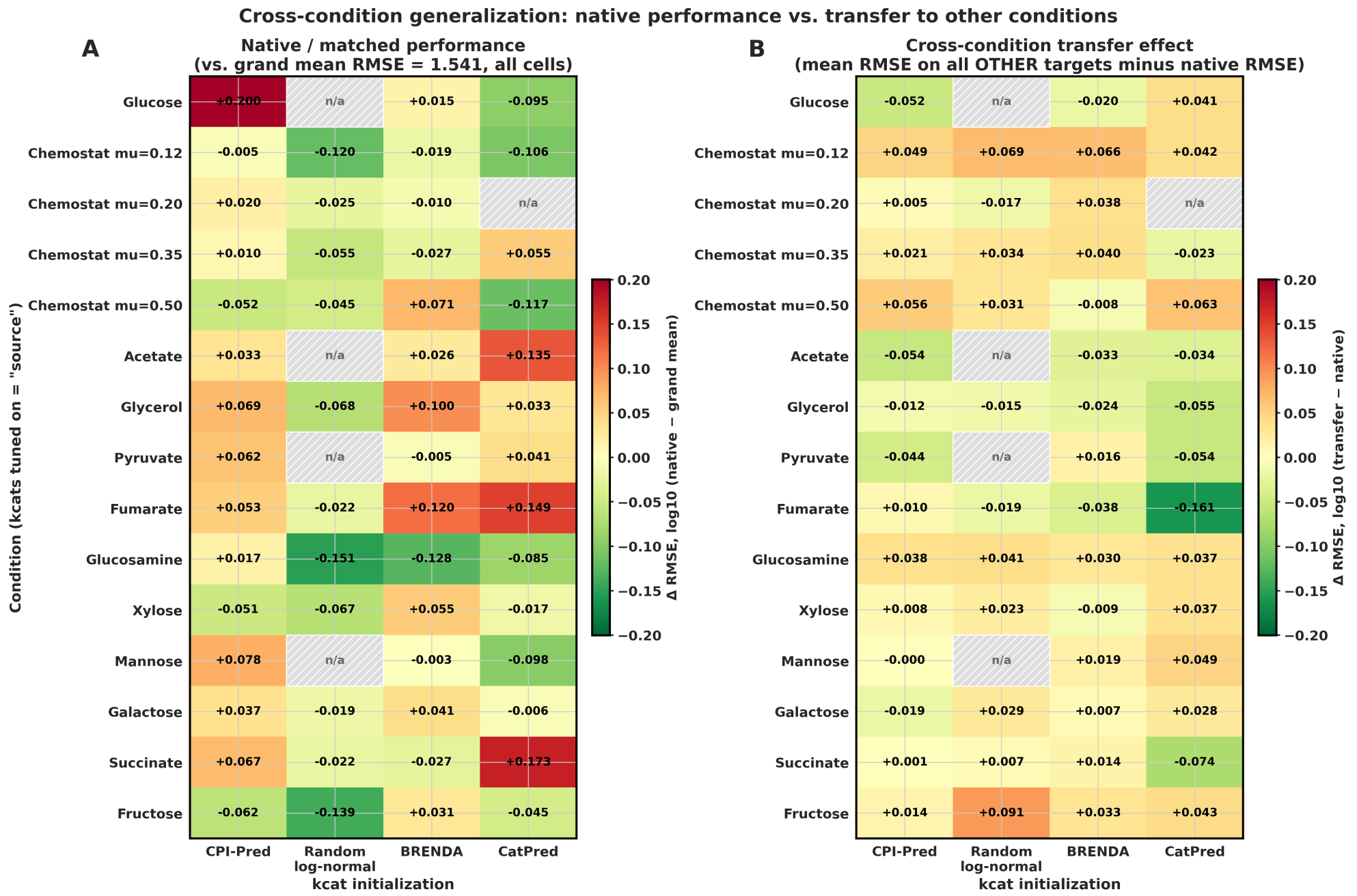

Fig D11: Cross-condition transfer of simulated-annealing-tuned k_{cat} vectors is comparable to native performance. **(A)** Native (matched-condition) RMSE against Schmidt et al. proteomics, expressed as a deviation from the grand mean RMSE across all 15 conditions $\times$ 4 k_{cat} sources (grand mean $= 1.541$, log10 scale). Red indicates native RMSE worse than the grand mean; green indicates better than the grand mean. **(B)** Cross-condition transfer effect: mean RMSE when a condition's tuned k_{cat} vector is applied to all other target conditions' media, minus that condition's own native RMSE. Values near zero (pale cells) indicate transfer performance comparable to native performance; red indicates worse transfer (unfavorable), green indicates better transfer (favorable) than native. Hatched cells (n/a) indicate condition-source combinations excluded due to non-convergent simulated annealing runs. The small magnitude and inconsistent sign of panel B across conditions and sources indicates no systematic overfitting of tuned k_{cat} vectors to their training condition.

Consistent with the main-text summary statistics (mean native $r = 0.336$ vs. mean cross-condition $r = 0.335$; Section 3.2), the transfer effect in panel B is small and inconsistently signed across conditions and k_{cat} sources (values clustering near zero, with roughly comparable numbers of positive and negative deltas), indicating no systematic degradation when k_{cat} vectors are applied outside their original tuning condition. A small number of condition-source pairs show larger transfer penalties

(e.g., Fumarate with CatPred, $\Delta = -0.161$ in the favorable direction; several Chemostat conditions show modest positive (i.e., unfavorable) transfer deltas in the +0.03 to +0.07 range), but no k_{cat} source or condition shows a consistent, large-magnitude generalization failure. Cells marked n/a correspond to condition-source combinations excluded due to non-convergent simulated annealing runs.

D.3 Condition constraints for *Rhodotorula toruloides* case study

Substrate uptake and O_2 consumption were imposed as maximum rates; CO_2 and reported by-products were allowed up to the measured secretion rates. On xylose, xylitol and D-arabinitol secretion were bounded by the measured rates; on acetate, citrate secretion was also bounded. Glucose, xylose, and acetate exchanges that were not the intended carbon source were blocked.

Table D3: Constraints for the *R. toruloides* reconstructions. Units: μ_{goal} in h^{-1}; E_{tot} and biomass fractions in g gDW^{-1}; uptake in mmol gDW^{-1} h^{-1}.

				Biomass			
Cond.	Carbon / N	μ_{goal}	E_{tot}	Prot.	Lip.	Carb.	Uptake
Gexp	glc / urea	0.191	0.08	0.64	0.05	0.20	2.489
GNlim	glc / urea	0.021	0.04	0.23	0.48	0.20	0.41
Xexp	xyl / NH_4^+	0.054	0.07	0.44	0.20	0.30	1.86
XNlim	xyl / NH_4^+	0.019	0.03	0.20	0.28	0.46	0.435
Aexp	ac / NH_4^+	0.073	0.09	0.39	0.18	0.37	6.1
ANlim	ac / NH_4^+	0.012	0.05	0.22	0.34	0.28	1.971

Table D4: Exchange bounds for the *R. toruloides* reconstructions.

	O_2	CO_2		By-products			
Cond.			N	glyc	xylitol	ara	cit
Gexp	−5.6	7.3	urea	0.049	—	—	—
GNlim	−1.16	1.440	urea	−0.007	—	—	—
Xexp	−2.678	2.845	NH_4^+	0	0.223	0.367	—
XNlim	−1.028	1.277	NH_4^+	0	0.004	0.077	—
Aexp	−7.144	5.224	NH_4^+	0	—	—	0.122
ANlim	−2.265	2.229	NH_4^+	0	—	—	−0.043

Appendix E Running kinGEMs on the BiGG Database

Table E5: Aggregate statistics for the kinGEMs pipeline applied across 93 completed BiGG genome-scale metabolic models spanning 23 organisms.

**Metric**	**Value**	**Range (min – max)**
Coverage		
Models attempted	94	—
Models completed	93	—
Organisms represented	23	—
Model size (per model, median)		
Unique reactions	3,731	155 – 9,879
Unique genes	1,304	137 – 1,766
CPI-Pred characterization (per model, median)		
Unique proteins mined	1,304	137 – 1,766
Rxn-gene pairs	11,127	541 – 32,817
CPI-Pred characterization (totals across all 93 models)		
Σ Proteins mined	103,900	—
Σ Rxn-gene pairs	885,282	—
k_{cat} *tuning (per model, median;* $n = 92$*)*		
Median initial k_{cat} (h^{-1})	20,708	7,119 – 31,322
Median tuned k_{cat} (h^{-1})	57,818	10,840 – 203,218
Median fold change	1.00	1.00 – 10.77
Simulated annealing biomass improvement ($n = 93$)		
Biomass improvement (%)	1,115	0 – 10,376

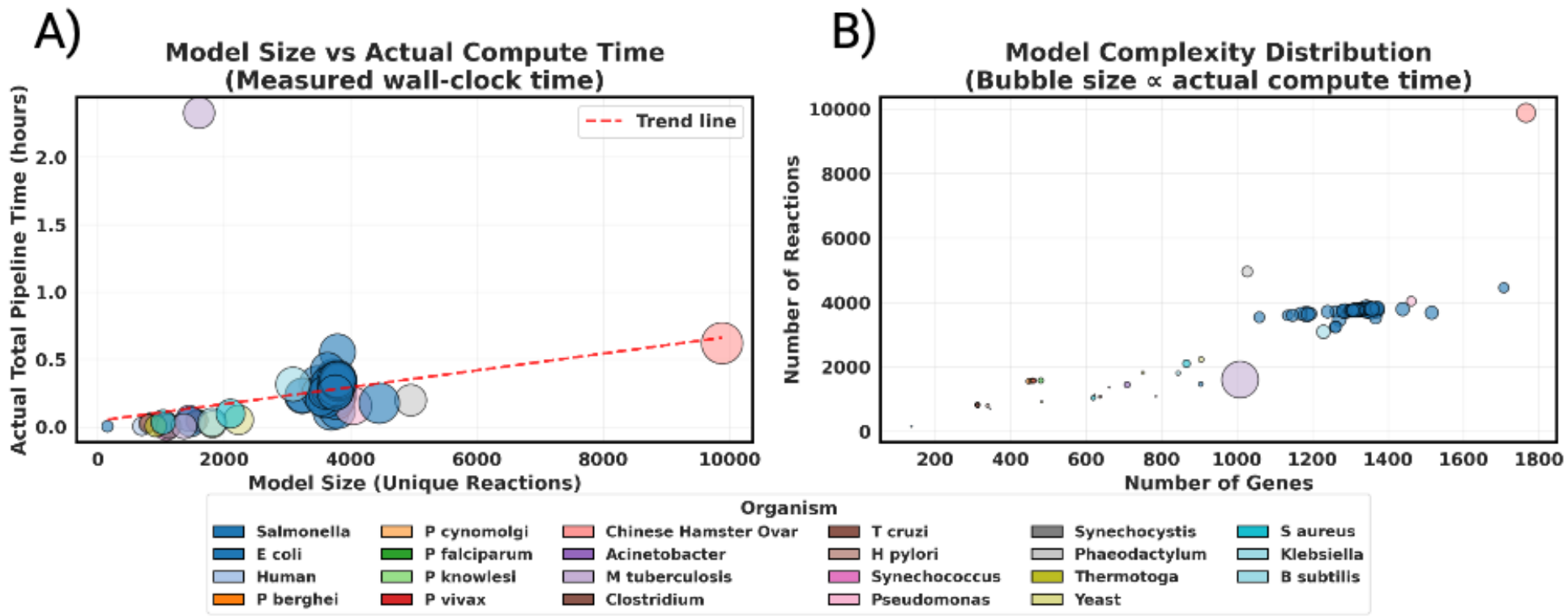

Fig E12: Computational scalability of the kinGEMs pipeline across 93 BiGG genome-scale metabolic models spanning 23 organisms. (A) Actual total pipeline wall-clock time as a function of model size (unique reactions) across all processed models. The dashed red trend line indicates a approximately linear scaling relationship between model size and compute time, with the majority of models completing in under 0.5 hours. The Chinese Hamster Ovary model, the largest model processed (9,800 reactions), required approximately 0.6 hours, demonstrating tractable runtime even for large-scale models. Bubble size is proportional to actual compute time. **(B)** Model complexity distribution across all processed organisms, with each bubble representing a model positioned by its number of genes (x-axis) and number of reactions (y-axis), and bubble size proportional to actual compute time. Models span a broad range of biological complexity, from small prokaryotic models (135 reactions) to large eukaryotic models (9,800 reactions), covering diverse taxa including bacteria, parasites, fungi, cyanobacteria, and mammalian cell lines.

Model	Organism	Rxns	Genes	Proteins (*CPI-Pred*)	$\tilde{k}_{cat}^{init}$	$\tilde{k}_{cat}^{tuned}$ (s^{-1})	$\Delta BM_{EC\ vs\ FBA}$ (%)
STM_v1_0 [4]	*S. enterica*	3,469	1,271	1,271	6.32	17.55	-0.9
e_coli_core [5]	*E. coli*	155	137	137	8.70	43.86	-2.0
iAB_RBC_283 [6]	Human (RBC)	698	346	346	3.45	3.69	0.0
iAF1260 [7]	*E. coli*	3,234	1,261	1,261	5.82	16.07	-8.4
iAF1260b [8]	*E. coli*	3,243	1,261	1,261	5.83	16.05	-8.4
iAM_Pb448 [9]	*P. berghei*	1,556	448	448	1.98	16.78	-13.6
iAM_Pc455 [9]	*P. cynomolgi*	1,565	455	455	2.21	8.86	-16.3
iAM_Pf480 [9]	*P. falciparum*	1,578	480	480	2.03	11.90	-15.4
iAM_Pk459 [9]	*P. knowlesi*	1,573	459	459	2.08	12.71	-13.7
iAM_Pv461 [9]	*P. vivax*	1,572	461	461	2.13	10.01	-12.1
iB21_1397 [10]	*E. coli*	3,770	1,337	1,337	5.76	13.24	-6.6
iBWG_1329 [10]	*E. coli*	3,775	1,329	1,329	5.74	16.22	-7.6
iCHOv1 [11]	CHO	9,879	1,766	1,766	2.25	4.58	0.0
iCN718 [12]	*A. baumannii*	1,447	709	709	5.68	10.17	-48.3
iE2348C_1286 [13]	*E. coli*	3,731	1,287	1,287	6.00	18.67	-6.6
iEC042_1314 [13]	*E. coli*	3,740	1,314	1,314	5.62	18.73	-6.9
iEC1344_C [13]	*E. coli*	3,691	1,344	1,344	—	—	0.0
iEC1349_Crooks [13]	*E. coli*	3,718	1,349	1,349	5.67	15.95	-7.5
iEC1356_Bl21DE3 [13]	*E. coli*	3,698	1,356	1,356	5.76	13.14	-10.4
iEC1364_W [13]	*E. coli*	3,721	1,364	1,364	5.89	16.07	-11.0
iEC1372_W3110 [13]	*E. coli*	3,722	1,372	1,372	5.76	15.30	-7.5
iEC55989_1330 [13]	*E. coli*	3,792	1,330	1,330	5.74	16.76	-7.7
iECABU_c1320 [13]	*E. coli*	3,762	1,320	1,320	5.87	17.67	-7.2
iECBD_1354 [13]	*E. coli*	3,777	1,354	1,354	5.81	13.14	-5.9
iECB_1328 [13]	*E. coli*	3,777	1,329	1,329	5.65	17.10	-7.2
iECDH10B_1368 [13]	*E. coli*	3,772	1,327	1,327	5.71	16.96	-8.6
iECDH1ME8569_1439 [13]	*E. coli*	3,790	1,439	1,439	5.71	15.30	-7.1
iECD_1391 [13]	*E. coli*	3,770	1,333	1,333	5.63	16.87	-6.9
iECED1_1282 [13]	*E. coli*	3,732	1,279	1,279	5.80	14.28	-6.9
iECH74115_1262 [13]	*E. coli*	3,718	1,262	1,262	5.68	14.72	-7.3
iECIAI1_1343 [13]	*E. coli*	3,795	1,343	1,343	5.81	17.29	-72.0
iECIAI39_1322 [13]	*E. coli*	3,751	1,321	1,321	5.69	17.15	-7.0
iECNA114_1301 [13]	*E. coli*	3,747	1,301	1,301	5.96	17.52	-6.6
iECO103_1326 [13]	*E. coli*	3,794	1,327	1,327	7.74	56.45	0.0
iECO111_1330 [13]	*E. coli*	3,793	1,328	1,328	5.64	13.91	-11.6
iECO26_1355 [13]	*E. coli*	3,814	1,355	1,355	5.64	13.14	-7.2
iECOK1_1307 [13]	*E. coli*	3,758	1,304	1,304	5.85	18.39	-6.4
iECP_1309 [13]	*E. coli*	3,769	1,309	1,309	5.98	18.11	-7.2
iECS88_1305 [13]	*E. coli*	3,756	1,305	1,305	5.85	17.70	-7.2
iECSE_1348 [13]	*E. coli*	3,804	1,348	1,348	5.84	17.54	-7.7
iECSF_1327 [13]	*E. coli*	3,773	1,327	1,327	5.93	16.72	-7.2
iECSP_1301 [13]	*E. coli*	3,736	1,299	1,299	5.86	18.62	-7.4
iECUMN_1333 [13]	*E. coli*	3,771	1,332	1,332	5.87	17.15	-7.3
iECW_1372 [13]	*E. coli*	3,815	1,372	1,372	5.77	14.41	-7.2
iECs_1301 [13]	*E. coli*	3,744	1,301	1,301	5.84	18.74	-7.9
iEK1008 [14]	*M. tuberculosis*	1,605	1,008	1,008	3.09	3.23	0.0
iEKO11_1354 [10]	*E. coli*	3,813	1,354	1,354	6.01	16.22	-7.1
iETEC_1333 [10]	*E. coli*	3,791	1,333	1,333	5.67	16.25	-8.1
iEcDH1_1363 [10]	*E. coli*	3,781	1,363	1,363	5.76	15.65	-7.4
iEcE24377_1341 [10]	*E. coli*	3,796	1,341	1,341	5.76	16.74	-7.1
iEcHS_1320 [10]	*E. coli*	3,784	1,321	1,321	5.63	17.30	-6.4
iEcSMS35_1347 [10]	*E. coli*	3,775	1,347	1,347	5.85	17.61	-6.6
iEcolC_1368 [10]	*E. coli*	3,799	1,368	1,368	5.68	15.93	-6.4
iG2583_1286 [10]	*E. coli*	3,730	1,283	1,283	5.87	19.94	-7.6
iHN637 [15]	*C. ljungdahlii*	1,077	637	637	5.50	6.43	0.0
iIS312	*T. cruzi*	826	312	312	2.92	3.76	-66.8
iIS312_Amastigote	*T. cruzi*	803	312	312	2.88	3.47	-74.5
iIS312_Epimastigote	*T. cruzi*	824	312	312	2.91	3.76	-66.8
iIS312_Trypomastigote	*T. cruzi*	810	312	312	2.84	3.01	-57.5
iIT341 [16]	H. pylori	796	339	339	4.68	6.07	-11.9
iJB785 [17]	Synechococcus	1,088	785	785	5.85	5.85	-0.0
iJN1463	Pseudomonas	4,038	1,462	1,462	4.89	5.72	-0.1
iJN678	Synechocystis	1,122	622	622	4.28	4.38	0.0
iJO1366 [18]	*E. coli*	3,518	1,367	1,367	5.74	15.05	-7.6
iJR904 [19]	*E. coli*	1,467	904	904	5.90	7.02	-45.9
iLB1027_lipid [20]	*P. tricornutum*	4,954	1,027	1,027	2.81	27.86	0.0
iLF82_1304 [10]	*E. coli*	3,759	1,302	1,302	6.04	17.76	-7.2
iLJ478 [21]	*T. maritima*	918	482	482	5.18	5.78	0.0
iML1515 [22]	*E. coli*	3,682	1,516	1,516	5.77	15.09	-10.8
iMM904 [23]	*S. cerevisiae*	2,230	905	905	4.08	4.55	-18.5
iND750 [24]	*S. cerevisiae*	1,815	750	750	4.11	4.78	0.0
iNJ661 [25]	*M. tuberculosis*	1,366	661	661	3.62	3.62	0.0
iNRG857_1313 [10]	*E. coli*	3,768	1,311	1,311	6.03	16.99	-6.4
iSB619 [26]	*S. aureus*	1,036	619	619	4.89	5.59	-99.3
iSBO_1134 [10]	*S. boydii*	3,597	1,134	1,134	5.99	15.35	0.0
iSDY_1059 [10]	*S. dysenteriae*	3,540	1,059	1,059	5.71	14.10	-13.9
iSFV_1184 [10]	*S. flexneri*	3,637	1,184	1,184	5.88	21.81	-14.6
iSF_1195 [10]	*S. flexneri*	3,648	1,195	1,195	5.91	25.54	-14.4
iSFxv_1172 [10]	*S. flexneri*	3,652	1,169	1,169	5.93	24.76	-15.7
iSSON_1240 [10]	*S. sonnei*	3,713	1,240	1,240	5.65	20.11	-8.0
iS_1188 [10]	*S. flexneri*	3,632	1,188	1,188	5.83	23.60	-16.9
iSbBS512_1146 [10]	*S. boydii*	3,599	1,147	1,147	5.72	30.72	-6.2
iUMN146_1321 [10]	*E. coli*	3,764	1,319	1,319	5.87	17.03	-6.3
iUMNK88_1353 [10]	*E. coli*	3,811	1,353	1,353	5.63	15.71	-7.4
iUTI89_1310 [10]	*E. coli*	3,754	1,310	1,310	5.83	17.14	-6.9
iWFL_1372 [10]	*E. coli*	3,815	1,372	1,372	5.77	14.44	-7.1
iY75_1357 [10]	*E. coli*	3,794	1,358	1,358	5.77	16.07	-7.4
iYL1228 [27]	*K. pneumoniae*	3,090	1,229	1,229	6.10	26.49	-10.2
iYO844 [28]	*B. subtilis*	1,809	844	844	4.70	6.33	0.0
iYS1720 [29]	*E. coli*	4,455	1,707	1,707	3.11	8.24	0.0
iYS854 [30]	*S. aureus*	2,101	866	866	5.37	6.43	-11.1
ic_1306 [10]	*E. coli*	3,756	1,307	1,307	5.99	18.17	-7.4
**Total / Median**		**3,731**†	**1,304**†	**103,900**‡	**5.75**†	**16.06**†	**-7.2**†

† Median across all 93 complete models. ‡ Sum across all 93 complete models. $\Delta BM_{EC\ vs\ FBA}$ = (final EC-model biomass − COBRA FBA biomass) / COBRA FBA biomass × 100%.

Table E6: Summary statistics across genome-scale metabolic models ($n = 93$ complete runs out of 94 attempted).

References

[1] Bynum, M. L. *et al.* *Pyomo - optimization modeling in Python, 3rd Edition* Vol. 67 (Springer, 2021).

[2] Crown, S. B., Long, C. P. & Antoniewicz, M. R. Integrated 13C-metabolic flux analysis of 14 parallel labeling experiments in Escherichia coli. *Metabolic Engineering* **28**, 151–158 (2015). URL https://doi.org/10.1016/j.ymben.2015.01.001.

[3] Schmidt, A. *et al.* The quantitative and condition-dependent Escherichia coli proteome. *Nature Biotechnology* **34**, 104–110 (2016). URL https://www.nature.com/articles/nbt.3418.

[4] Thiele, I. *et al.* A community effort towards a knowledge-base and mathematical model of the human pathogen Salmonella Typhimurium LT2. *BMC systems biology* **5**, 8 (2011).

[5] Orth, J. D., Fleming, R. M. T. & Palsson, B. O. Reconstruction and Use of Microbial Metabolic Networks: the Core Escherichia coli Metabolic Model as an Educational Guide. *EcoSal Plus* **4**, 10.1128/ecosalplus.10.2.1 (2010). URL https://journals.asm.org/doi/10.1128/ecosalplus.10.2.1.

[6] Bordbar, A., Jamshidi, N. & Palsson, B. O. iAB-RBC-283: A proteomically derived knowledge-base of erythrocyte metabolism that can be used to simulate its physiological and patho-physiological states. *BMC systems biology* **5**, 110 (2011).

[7] Feist, A. M. *et al.* A genome-scale metabolic reconstruction for Escherichia coli K-12 MG1655 that accounts for 1260 ORFs and thermodynamic information. *Molecular Systems Biology* **3**, 121 (2007).

[8] Feist, A. M. *et al.* Model-driven evaluation of the production potential for growth-coupled products of *Escherichia coli*. *Metabolic Engineering* **12**, 173–186 (2010). URL https://www.sciencedirect.com/science/article/pii/S1096717609000895.

[9] Abdel-Haleem, A. M. *et al.* Functional interrogation of Plasmodium genus metabolism identifies species- and stage-specific differences in nutrient essentiality and drug targeting. *PLoS computational biology* **14**, e1005895 (2018).

[10] Monk, J. M. *et al.* Genome-scale metabolic reconstructions of multiple Escherichia coli strains highlight strain-specific adaptations to nutritional environments. *Proceedings of the National Academy of Sciences of the United States of America* **110**, 20338–20343 (2013).

[11] Hefzi, H. *et al.* A Consensus Genome-scale Reconstruction of Chinese Hamster Ovary Cell Metabolism. *Cell Systems* **3**, 434–443.e8 (2016).

[12] Norsigian, C. J., Kavvas, E., Seif, Y., Palsson, B. O. & Monk, J. M. iCN718, an Updated and Improved Genome-Scale Metabolic Network Reconstruction of Acinetobacter baumannii AYE. *Frontiers in Genetics* **9**, 121 (2018).

[13] Monk, J. M. *et al.* Multi-omics Quantification of Species Variation of Escherichia coli Links Molecular Features with Strain Phenotypes. *Cell Systems* **3**, 238–251.e12 (2016).

[14] Kavvas, E. S. *et al.* Updated and standardized genome-scale reconstruction of Mycobacterium tuberculosis H37Rv, iEK1011, simulates flux states indicative of physiological conditions. *BMC systems biology* **12**, 25 (2018).

[15] Nagarajan, H. *et al.* Characterizing acetogenic metabolism using a genome-scale metabolic reconstruction of Clostridium ljungdahlii. *Microbial Cell Factories* **12**, 118 (2013).

[16] Thiele, I., Vo, T. D., Price, N. D. & Palsson, B. O. Expanded metabolic reconstruction of Helicobacter pylori (iIT341 GSM/GPR): an in silico genome-scale characterization of single- and double-deletion mutants. *Journal of Bacteriology* **187**, 5818–5830 (2005).

[17] Broddrick, J. T. *et al.* Unique attributes of cyanobacterial metabolism revealed by improved genome-scale metabolic modeling and essential gene analysis. *Proceedings of the National Academy of Sciences of the United States of America* **113**, E8344–E8353 (2016).

[18] Orth, J. D. *et al.* A comprehensive genome-scale reconstruction of Escherichia coli metabolism–2011. *Molecular Systems Biology* **7**, 535 (2011).

[19] Reed, J. L., Vo, T. D., Schilling, C. H. & Palsson, B. O. An expanded genome-scale model of Escherichia coli K-12 (iJR904 GSM/GPR). *Genome Biology* **4**, R54 (2003).

[20] Levering, J. *et al.* Genome-Scale Model Reveals Metabolic Basis of Biomass Partitioning in a Model Diatom. *PloS One* **11**, e0155038 (2016).

[21] Zhang, Y. *et al.* Three-dimensional structural view of the central metabolic network of Thermotoga maritima. *Science* **325**, 1544–1549 (2009).

[22] Monk, J. M. *et al.* iML1515, a knowledgebase that computes Escherichia coli traits. *Nature biotechnology* **35**, 904–908 (2017). URL https://www.ncbi.nlm.nih.gov/pmc/articles/PMC6521705/.

[23] Mo, M. L., Palsson, B. O. & Herrgård, M. J. Connecting extracellular metabolomic measurements to intracellular flux states in yeast. *BMC systems biology* **3**, 37 (2009).

[24] Duarte, N. C., Herrgård, M. J. & Palsson, B. O. Reconstruction and validation of Saccharomyces cerevisiae iND750, a fully compartmentalized genome-scale metabolic model. *Genome Research* **14**, 1298–1309 (2004).

[25] Jamshidi, N. & Palsson, B. O. Investigating the metabolic capabilities of Mycobacterium tuberculosis H37Rv using the in silico strain iNJ661 and proposing alternative drug targets. *BMC systems biology* **1**, 26 (2007).

[26] Becker, S. A. & Palsson, B. O. Genome-scale reconstruction of the metabolic network in Staphylococcus aureus N315: an initial draft to the two-dimensional annotation. *BMC microbiology* **5**, 8 (2005).

[27] Liao, Y.-C. *et al.* An experimentally validated genome-scale metabolic reconstruction of Klebsiella pneumoniae MGH 78578, iYL1228. *Journal of Bacteriology* **193**, 1710–1717 (2011).

[28] Oh, Y.-K., Palsson, B. O., Park, S. M., Schilling, C. H. & Mahadevan, R. Genome-scale reconstruction of metabolic network in Bacillus subtilis based on high-throughput phenotyping and gene essentiality data. *The Journal of Biological Chemistry* **282**, 28791–28799 (2007).

[29] Seif, Y. *et al.* Genome-scale metabolic reconstructions of multiple Salmonella strains reveal serovar-specific metabolic traits. *Nature Communications* **9**, 3771 (2018).

[30] Seif, Y. *et al.* A computational knowledge-base elucidates the response of Staphylococcus aureus to different media types. *PLoS computational biology* **15**, e1006644 (2019).
